# Genome analysis of five recently described species of the CUG-Ser clade uncovers *Candida theae* as a new hybrid lineage with pathogenic potential in the *Candida parapsilosis* species complex

**DOI:** 10.1093/dnares/dsac010

**Published:** 2022-04-19

**Authors:** Verónica Mixão, Valentina del Olmo, Eva Hegedűsová, Ester Saus, Leszek Pryszcz, Andrea Cillingová, Jozef Nosek, Toni Gabaldón

**Affiliations:** 1 Life Sciences Department, Barcelona Supercomputing Center (BSC), 08034 Barcelona, Spain; 2 Mechanisms of Disease Department, Institute for Research in Biomedicine (IRB), Barcelona, Spain; 3 Department of Biochemistry, Faculty of Natural Sciences, Comenius University in Bratislava, 842 15 Bratislava, Slovak Republic; 4 Centre for Genomic Regulation (CRG), The Barcelona Institute of Science and Technology, Barcelona 08003, Spain; 5 ICREA, Barcelona 08010, Spain; 6 Centro de Investigación Biomédica En Red de Enfermedades Infecciosas, Barcelona, Spain

**Keywords:** *Candida parapsilosis* clade, *Candida* yeast hybrids, genome sequence, linear mitochondrial genome, hydroxyaromatic compounds

## Abstract

*Candida parapsilosis* species complex comprises three important pathogenic species: *Candida parapsilosis sensu stricto*, *Candida orthopsilosis* and *Candida metapsilosis*. The majority of *C. orthopsilosis* and all *C. metapsilosis* isolates sequenced thus far are hybrids, and most of the parental lineages remain unidentified. This led to the hypothesis that hybrids with pathogenic potential were formed by the hybridization of non-pathogenic lineages that thrive in the environment. In a search for the missing hybrid parentals, and aiming to get a better understanding of the evolution of the species complex, we sequenced, assembled and analysed the genome of five close relatives isolated from the environment: *Candida jiufengensis*, *Candida pseudojiufengensis*, *Candida oxycetoniae*, *Candida margitis* and *Candida theae*. We found that the linear conformation of mitochondrial genomes in *Candida* species emerged multiple times independently. Furthermore, our analyses discarded the possible involvement of these species in the mentioned hybridizations, but identified *C. theae* as an additional hybrid in the species complex. Importantly, *C. theae* was recently associated with a case of infection, and we also uncovered the hybrid nature of this clinical isolate. Altogether, our results reinforce the hypothesis that hybridization is widespread among *Candida* species, and potentially contributes to the emergence of lineages with opportunistic pathogenic behaviour.

## 1. Introduction

*Candida* species are a non-monophyletic group of yeasts that include important human pathogens.^1^ Although the majority of *Candida* infections (candidiasis) are caused by *Candida albicans*, *Candida glabrata* and *Candida parapsilosis*, the epidemiology of this disease is shifting with the emergence of other pathogenic lineages.^1^^,^^2^ The mechanisms involved in the emergence of pathogenicity are still unknown, but hybridization has been suggested as a possible evolutionary path for the appearance of novel yeast pathogens.^3^^,^^4^ Indeed, in the *C. parapsilosis* species complex, at least six natural independent hybridization events have occurred, leading to four *C. orthopsilosis* and two *C. metapsilosis* hybrid lineages with the potential to infect humans.^5–8^ While one of the two parental lineages for *C. orthopsilosis* hybrids has already been isolated, also in the clinical setting, for *C. metapsilosis* none of the three inferred parentals has been found thus far.^6–8^ The absence of these parentals from clinical isolates has led to the hypothesis that they are possibly non-pathogenic and may thrive in the environment, and that these hybridization events contributed to the emergence of the pathogenic lineages identified as a source for candidiasis.^3^^,^^6^

Although most research studies tend to focus on *Candida* clinical isolates, *Candida* species, including the strains affiliated to the *C. parapsilosis* species complex, are commonly isolated from various environmental niches, such as soil, plants and the digestive tract of phytophagous insects. Therefore, it is not surprising that these yeasts are able to assimilate various simple aromatic compounds originating from lignin decomposition in decaying plant tissues.^9^ Aromatic compounds, such as hydroxybenzenes and hydroxybenzoates, are metabolized in the 3-oxoadipate or in the gentisate pathways. However, even closely related species can grow on different hydroxyaromatic substrates and vary in their inventory of corresponding biochemical pathways. For example, *C. parapsilosis* utilizes dihydroxybenzenes (hydroquinone, resorcinol), mono- and dihydroxybenzoates (4-hydroxybenzoate, 2,4-dihydroxybenzoate, protocatechuate) in the hydroxyhydroquinone (HHQ) variant of the 3-oxoadipate pathway. In addition, this species also metabolizes 3-hydroxybenzoate and gentisate in the gentisate pathway. In contrast, the growth of the other two members of the *C. parapsilosis* species complex (*C. orthopsilosis* and *C. metapsilosis*) on 2,4-dihydroxybenzoate and the gentisate pathway substrates is impaired. Similarly, *C. albicans* does not possess the gentisate pathway and it is unable to assimilate hydroxybenzoates. However, this yeast metabolizes phenol and dihydroxybenzenes including catechol using two branches (catechol and a shorter version of HHQ) of the 3-oxoadipate pathway.^10^^,^^11^ Recently, another CUG-Ser clade species, *Candida subhashii*, has been shown to possess the gentisate pathway as well as both branches of the 3-oxoadipate pathway, which likely corresponds to the ancestral inventory of biochemical pathways for hydroxyaromatic substrate utilization.^12^

Another peculiarity of the *C. parapsilosis* species complex is the molecular architecture of the mitochondrial DNA. Unlike most other yeasts, *C. parapsilosis*, *C. metapsilosis* and *C. orthopsilosis* contain a linear mitochondrial genome,^13^^,^^14^ although circularized mutants formed by end-to-end fusions of the linear DNA molecules were identified in several strains of *C. metapsilosis* and *C. orthopsilosis.*^6^^*,*^^15–17^ The linear mitochondrial DNA molecules terminate at both ends with arrays of long tandem repeats (i.e. n × 738 bp in *C. parapsilosis sensu stricto*^18^^*,*^^19^). These mitochondrial telomeres are replicated via a telomerase-independent pathway, reminding the alternative mechanism of telomere maintenance operating in a subset of human cancer and immortal cell lines.^20^

*Candida jiufengensis*, *Candida pseudojiufengensis*, *Candida oxycetoniae*, *Candida margitis* and *Candida theae* are five recently described species isolated from the environment. While *C. jiufengensis, C. pseudojiufengensis*, *C. oxycetoniae* and *C. margitis* were isolated from the gut of flower beetles in China,^21–23^*C. theae* was described from isolates obtained from modern tea drinks and ancient chicha fermentation vessels in Taiwan and Ecuador, respectively.^23^ As preliminary phylogenetic analyses placed all of these species as close relatives to the *C. parapsilosis* species complex,^21–23^ we hypothesized that some could correspond—or be closely related—to the missing parental lineages of the above-mentioned hybrids. Moreover, we considered that their study could provide additional insight into the metabolic diversity of the *C. parapsilosis* species complex and into the evolutionary emergence of the linear genome in mitochondria and the mechanism for maintenance of its telomeres. As none or their genomes were available at the start of this project, we sequenced, assembled and analysed the genome of the type strains of these species. Our results showed that these lineages do not correspond to any of the parentals of *C. orthopsilosis* and *C. metapsilosis* hybrids but, interestingly, revealed an additional hybrid lineage in the *C. parapsilosis* species complex: *C. theae*, which was recently shown to have pathogenic potential.^24^ We also show that similarly to *C.metapsilosis*, *C. orthopsilosis* and *C. parapsilosis*, the mitochondrial genomes of *C. margitis* and *C. theae* are linear and terminate with arrays of long tandem repeats making this mitochondrial genome architecture a typical feature of the *C. parapsilosis* species complex.

## 2. Materials and methods

### 2.1. Genomic DNA sequencing

Genomic DNA paired-end sequencing was performed for the type strain of *C. jiufengensis* (CBS10846)*, C. pseudojiufengensis* (CBS10847), *C. oxycetoniae* (CBS10844)*, C. margitis* (CBS14175) and *C. theae* (CBS12239), as described in Mixão et al.^12^ For *C. theae* a mate-pair library was also sequenced. To this end, DNA was fragmented to sizes between 1 and 20 kb using a transposase that binds biotinylated adapters at the breaking point. Strand displacement was performed to ‘repair’ the nicks left by the transposase. Fragment sizes of 3–6 kb were then selected on a 0.8% agarose gel and were then circularized. Non-circularized DNA was removed by digestion. The circular DNA was then mechanically sheared to fragments of ∼100 bp to 1 kb and the fragments containing the biotinylated ends were pulled down using magnetic streptavidin beads and submitted to a standard library preparation. A final size selection on 2% agarose gel was done and fragments of 400–700 bp were selected for the final library. Final libraries were analysed using Agilent High Sensitivity chip to estimate the quantity and check size distribution and were then quantified by qPCR using the KAPA Library Quantification Kit (ref. KK4835, Kapa Biosystems) prior to amplification with Illumina’s cBot. Libraries were sequenced 2 × 125 bp on Illumina’s HiSeq 2500.

### 2.2. *Candida theae* public data

For the analysis of the *C. theae* clinical isolate, publicly available data were retrieved from the NCBI database. Specifically, we downloaded the Illumina raw sequencing reads and the genome assembly under the accession number PRJNA600777.^24^ For *k*-mer comparisons to assess possible parental relationships, sequencing libraries of *C. orthopsilosis* (ERR380554) and *C. metapsilosis* (ERR247393) were also downloaded.^5^^,^^6^

### 2.3. Genome assembly

Raw sequencing data from all studied strains were inspected with FastQC v0.11.5 (http://www.bioinformatics.babraham.ac.uk/projects/fastqc/). Paired end reads were filtered for quality below 10 or size below 31 bp and for the presence of adapters with Trimmomatic v0.36.^25^ Mate-pair reads were filtered with NxTrim v0.4.1-53c2193.^26^ After filtering, only reads clearly identified as paired-end or mate-pair by the respective programs were used for subsequent analysis. The K-mer Analysis Toolkit (KAT^27^) was used to count *k-*mer frequency and estimate the expected genome size. SOAPdenovo v2.04^28^ and SPAdes v3.9 in both SPAdes and dipSPAdes modes^29^^,^^30^ were used separately to perform the genome assembly. Redundant contigs were removed with Redundans v0.13c.^31^ The quality of the different assemblies was inspected with Quast v4.5.^32^ Genome annotation was performed with Augustus v3.5 using *C. albicans* as model organism.^33^ KAT and BUSCO v3 (Ascomycota database)^27^^,^^34^ were used to estimate the assembly completeness. The best assembly for each species was chosen based on the assembly completeness, genome size, N50 and number of scaffolds. Functional annotation was performed with eggNOG-mapper web-server using the default settings.^35^

### 2.4. Read mapping and variant calling

Read mapping was performed with BWA-MEM v0.7.15.^36^ Picard v2.1.1 (http://broadinstitute.github.io/picard/) was used to sort the resultant file by coordinate, as well as, to mark duplicates, create the index file and obtain the mapping statistics. The mapping was inspected with IGV version 2.0.30.^37^ Mapping coverage was determined with SAMtools v0.1.18.^38^

Samtools v0.1.18^38^ and Picard v2.1.1 (http://broadinstitute.github.io/picard/) were used to index the reference and create its dictionary, respectively, for posterior variant calling. GATK v3.6^39^ was used to call variants with HaplotypeCaller set with –genotyping_mode DISCOVERY -stand_emit_conf 10 -stand_call_conf 30 -ploidy 2 -nct 8. The tool VariantFiltration of the same programme was used to filter the vcf files with the following parameters: –clusterSize 5 –clusterWindowSize 20 –genotypeFilterName ‘heterozygous’ –genotypeFilterExpression ‘isHet == 1’ –filterName ‘bad_quality’ -filter ‘QD < 2.0 ‖ MQ < 40 ‖ FS > 60.0 ‖ HaplotypeScore > 13.0 ‖ MQRankSum < -12.5 ‖ ReadPosRankSum < -8.0’ –filterExpression ‘DP <= 20’ –filterName ‘DepthofQuality’. In order to determine the number of Single Nucleotide Polymorphisms per Kilobase (SNPs/kb), a file containing only SNPs was generated with the SelectVariants tool. Moreover, for this calculation, only positions in the reference with 20 or more reads were considered for the genome size, and these were determined with bedtools genomecov v2.25.0.^40^ Ploidy estimation was calculated with nQuire histotest.^41^ Average depth of coverage was calculated with Mosdepth v0.3.1.^42^

### 2.5. Loss of heterozygosity block definition

To determine for each heterozygous strain the presence of loss of heterozygosity (LOH) blocks, heterozygous and homozygous variants were separated. Then, the procedure applied and validated by Pryszcz et al.^6^ was used. Briefly, bedtools merge v2.25.0^40^ with a window of 100 bp was used to define heterozygous regions, and by opposite, LOH blocks would be all non-heterozygous regions in the genome. Minimum heterozygous and LOH block size was established at 100 bp.

### 2.6. Phylome reconstruction

PhylomeDB pipeline^43^ was used for phylome reconstruction as described in Mixão et al.,^44^ considering the 28 species specified in Supplementary File S1, and using each of the five target species of this study as seed. Gene gain and loss analysis in the seed branch was performed based on the phylome results. An enrichment analysis was done using FatiGO.^45^ Species-tree reconstruction was based on the final concatenated alignment of 460 single genes, comprising 283,609 amino-acid positions, with RAxML v8.2.4,^46^ using the PROTGAMMALG substitution model. Gene phylogenies were used to determine the orthologs of *C. albicans* and *C. parapsilosis* genes involved in metabolic pathways.

### 2.7. MAT locus analysis

The MAT locus alleles of *C. orthopsilosis* were retrieved from the reference genome assembly (PRJEA172256).^47^ A BLASTp using these genes as a query was performed on a database containing the predicted protein-coding genes of *C. theae* and *C. margitis*. A ‘reciprocal best-hit’ approach was used to confidently identify the MAT locus of these species.

### 2.8. Mitochondrial DNA analysis

For mitochondrial DNA analysis, we retrieved the mitochondrial sequences available at NCBI for *C. jiufengensis* (GU136397^48^), *C. pseudojiufengensis* (KC993179^49^), *C. oxycetoniae* (KC993187^49^) and *C. theae* (KC993195^49^). The mitochondria of *C. margitis* was assembled in this study with NOVOPlasty v4.2^50^ using *C. parapsilosis* COX2 as seed (NC_005253.2: c8960-8166). The mitochondrial DNA sequences were annotated using the MFannot tool (http://megasun.bch.umontreal.ca/cgi-bin/mfannot/mfannotInterface.pl) and manually curated with the Geneious package (version R 11.1.5). Terminal sequences (mitochondrial telomeres) of the type 2 linear mitochondrial DNA were symmetrically reconstructed on both sides of the mitochondrial contigs according to the model of *C. parapsilosis* mitochondrial DNA.^18^^,^^19^ OGDRAW (https://chlorobox.mpimp-golm.mpg.de/OGDraw.html^51^) was used for visualization of the mitochondrial genome maps.

### 2.9. Yeast strains and carbon substrate assimilation tests

The type strains of *C. jiufengensis* AS 2.3688 (CBS10846)*, C. pseudojiufengensis* AS 2.3693 (CBS10847), *C. oxycetoniae* AS 2.3656 (CBS10844), *C. margitis* (CBS14175) and *C. theae* G-17 (CBS12239) were investigated in this study. In addition, for the screening of hydroxyaromatic substrate assimilation, we also used the type strains of *C. parapsilosis* CLIB214 (CBS604), *C. orthopsilosis* MCO457 (CBS10906), *C. metapsilosis* MCO448 (CBS10907) and the reference strain of *C. albicans* SC5314. In these tests, the yeast cultures were grown overnight at 28°C in complex liquid media [1% (w/v) yeast extract, 2% (w/v) peptone] containing 2% (w/v) glucose or 1.5% (v/v) glycerol (for cells spotted on the medium with resorcinol). The cells were washed with water and the diluted suspensions were spotted onto the plates with synthetic media [0.17% (w/v) Yeast Nitrogen Base without Amino Acids and Ammonium Sulphate (Difco), 0.5% (w/v) ammonium sulphate, 2% (w/v) agar] containing either 2% (w/v) glucose or 10 mM hydroxyaromatic compound as a sole carbon source [i.e. 3-hydroxybenzoate, gentisate (2,5-dihydroxybenzoate), phenol, catechol (1,2-dihydroxybenzene), 4-hydroxybenzoate, 2,4-dihydroxybenzoate, protocatechuate (3,4-dihydroxybenzoate), hydroquinone (1,4-dihydroxybenzene) and resorcinol (1,3-dihydroxybenzene)]. Hydroxyaromatic compounds were dissolved in dimethyl sulphoxide as 0.5 M stocks prior addition to the medium.

### 2.10. Data availability

Sequencing reads, genome assemblies and the respective annotation of *C. jiufengensis* (CBS10846)*, C. pseudojiufengensis* (CBS10847), *C. oxycetoniae* (CBS10844)*, C. margitis* (CBS14175) and *C. theae* (CBS12239) are available at NCBI database under the BioProject accessions PRJNA726921, PRJNA726922, PRJNA726927, PRJNA748886 and PRJNA726928, respectively. The phylomes of these species, and the corresponding orthology and paralogy relationships, are available for browsing or download at PhylomeDB^43^ with the IDs: 16 (*C. jiufengensis*), 404 (*C. pseudojiufengensis*), 960 (*C. oxycetoniae*), 423 (*C. margitis*) and 866 (*C. theae*).

## 3. Results and discussion

### 3.1. Genome assembly and phylome reconstruction

In this study, we performed whole-genome sequencing, assembly and annotation of the type strains of *C. jiufengensis* (CBS10846)*, C. pseudojiufengensis* (CBS10847), *C. oxycetoniae* (CBS10844)*, C. margitis* (CBS14175) and *C. theae* (CBS12239). We applied different assembly strategies in parallel (see Materials and methods), and the final assembly for each species was chosen based on several quality parameters, such as genome completeness, percentage of mapped reads, N50 and assembly fragmentation. The metrics of the final assembly for each species are summarized in Table 1. Importantly, estimations based on predicted protein-coding genes and *k-*mer composition reported > 94% and > 99% assembly completeness, respectively, for each of the genomes (Table 1), thus showing that these are good representatives of the respective isolate and can be used for further analysis.

**Table 1:** Summary of the assembly metrics for the analysed species of the CUG-Ser clade, namely, *C. jiufengensis, C. pseudojiufengensis, C. oxycetoniae, C. margitis* and *C. theae* information on expected and observed genome sizes, number of contigs, N50, GC content, assembly completeness, number of predicted protein-coding genes, mapped reads, genomic variability and assembly strategy are provided

	*C. jiufengensis*	*C. pseudojiufengensis*	*C. oxycetoniae*	*C. margitis*	*C. theae*
Estimated size^a^ (Mb)	13.83	15.98	11.31	14.37	12.36
Assembly size (Mb)	13.70	14.03	12.82	12.93	12.45
Contigs	22	56	194	189	181
Contigs >50 kb	15	31	85	67	141
N50	1,273,379	828,659	149,712	193,096	205,21
GC	27.22%	28.22%	37.76%	38.51%	40.23%
Assembly completeness (KAT)	99.94%	99.68%	99.83%	99.76%	99.25%
Proteome completeness (BUSCO)	98.90%	99.10%	96.00%	94.2%	97.50%
Protein-coding genes	5,673	5,814	4,920	5,551	5,407
Mapped reads	99.88%	99.43%	96.99%	99.45%	99.35%
SNPs/kb (heterozygous)	0.03 (0.02)	0.09 (0.09)	0.07 (0.04)	0.30 (0.30)	6.36 (6.34)
Parental divergence	—	—	—	—	3.15%
LOH	—	—	—	—	84.41%
Assembly strategy	SOAPdenovo + Redundans	dipSPAdes + Redundan	SOAPdenovo + Redundans	SOAPdenovo + Redundans	SPAdes + Redundans

a Estimated with KAT.^27^

**Figure 1:**
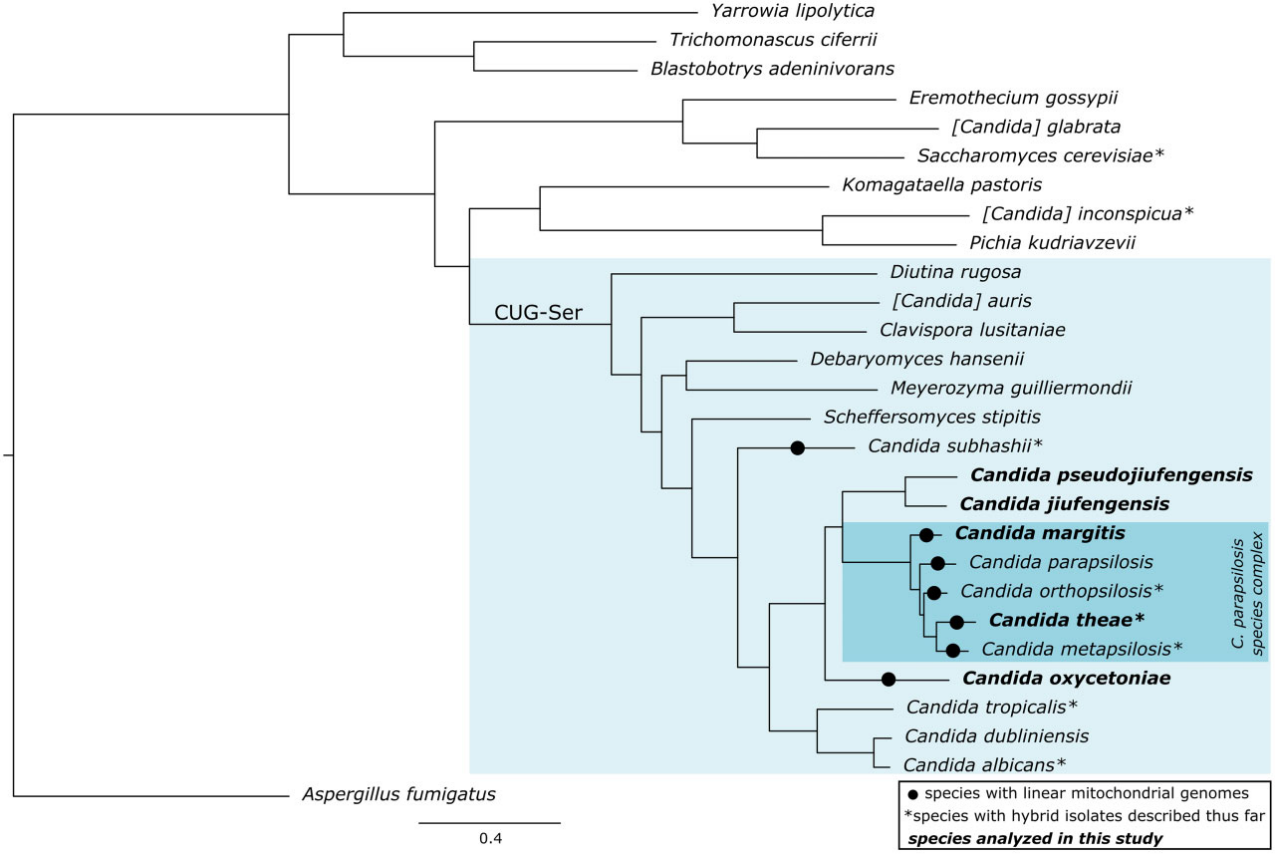
Maximum-likelihood tree reconstruction of the concatenated alignment of the 460 protein-coding genes shared by the 28 species used for phylome reconstruction. Species where hybrid strains have been isolated are marked with asterisk (*). The five species analysed in this study are in bold. Circles indicate species with linear mitochondrial genomes.

We next reconstructed the phylome (i.e. the complete collection of gene phylogenies^52^) for each of the five target species in the context of 23 additional ones (Fig. 1, Supplementary File S1). Our results revealed the existence of 194, 282, 221, 109 and 33 orphan genes in *C. jiufengensis, C. pseudojiufengensis, C. oxycetoniae, C. theae* and *C. margitis*, respectively. Functional enrichment analyses revealed that genes specifically duplicated in *C. jiufengensis* are enriched in aspartic-type endopeptidase activity and transmembrane transport activity, while those specifically duplicated in *C. pseudojiufengensis* are enriched in aspartic-type endopeptidase activity, and the ones specifically duplicated in *C. margitis* are enriched in transmembrane transport activity (Supplementary File S2). No enrichment was observed in *C. oxycetoniae* and *C. theae*. The species tree obtained (Fig. 1, see Materials and Methods for details) shows that, although being close-relatives, *C. jiufengensis, C. pseudojiufengensis* and *C. oxycetoniae* are not part of the *C. parapsilosis* species complex, and hence they cannot correspond to any of the parental lineages of the above-mentioned hybrids. By contrast, *C. theae* and *C. margitis* are closely related to *C. orthopsilosis* and *C. metapsilosis* and further analysis was necessary to assess their putative role in the hybridizations. The genome-wide collections of gene phylogenies and corresponding alignments, as well as the corresponding orthology and paralogy predictions with other species, constitute a valuable resource for future investigations of these species and are available for browsing or download at PhylomeDB and MetaPhORs databases.^43^^,^^53^

### 3.2. *K*-mer frequency and genomic variability

We analysed the *k-*mer frequency patterns of each of these genomes (see Materials and methods). In this type of analysis, the *k*-mer frequency across read coverage is used as an indicator of the amount of genomic variability. For instance, when a haploid or highly homozygous genome is analysed, all the *k*-mers have similar coverage and are expected to be represented in the assembly, which is translated as a single density peak in the *k*-mer plot. On the other hand, when a highly heterozygous diploid genome is analysed, such a plot presents two peaks, as one corresponds to regions where both chromosomes have the same *k*-mer, and the other one to the regions where both chromosomes differ (two different *k*-mers, resulting in half coverage for each). Moreover, in this last scenario, if the assembly includes only one of the haplotypes of the heterozygous regions (as it is the case of the assemblies of this study), we expect that only half of the *k*-mers of the heterozygous peak are represented in the assembly.^54^ Our results show that *C. jiufengensis, C. pseudojiufengensis* and *C. oxycetoniae* have a single *k-*mer frequency peak, while *C. margitis* and *C. theae* seem to have two (Supplementary Fig. S1). Consistently with this finding, we identified heterozygous SNPs in all five species, but at a much higher level in the last two (Table 1). These findings support a diploid nature for all the five type isolates and they also suggest that *C. margitis* and *C. theae* are highly heterozygous and present a higher genomic variability as compared with the other species. Of note, the *k*-mer comparison between the five species analysed in this study and the hybrids described in the *C. parapsilosis* species complex, did not reveal any shared *k*-mer, completely excluding their role in the hybridization events previously reported in this clade.

To obtain a better understanding of the heterozygous nature of *C. margitis* and *C. theae*, we performed a closer inspection of their genomes. Contrary to what was expected for a diploid configuration, the *k*-mer patterns of *C. margitis* revealed that the first peak represents four-fifths of the coverage of the second one, and that all the *k*-mers are present in the assembly (Supplementary Fig. S1). Moreover, the attempt to determine the ploidy of this species with nQuire^41^ was inconclusive (nQuire histotest *r*^2^ = 0.50). Altogether, these results suggest that *C. margitis* harbours chromosomes at different copy numbers and not highly heterozygous ones, otherwise, the process of removing redundant contigs would have led to the absence of some *k-*mers from the assembly. To confirm this hypothesis, we inspected the average coverage of each scaffold. Although we found that indeed some scaffolds have much higher read coverage than the others, the proportion of their coverage was not consistent with the proportion found in the *k-*mer frequency analysis (Supplementary Fig. S2). Therefore, we are uncertain whether these two peaks in the *k*-mer plot may represent real copy number variations among *C. margitis* chromosomes, or a sequencing artefact resulting in coverage irregularities.

Regarding the *k-mer* profile of *C. theae*, the two peaks of the *k-mer* plot are clearly associated to a diploid genome because: (i) the first peak has half coverage of the second one, and (ii) half of the *k-*mers of the first peak (heterozygous) are absent of the assembly (Supplementary Fig. S1), consistently with the assembly reduction that we expect for a diploid genome. Indeed, our estimates suggest that *C. theae* is diploid (nQuire histotest *r*^2^ = 0.97), and the genomic variability of *C. theae* type strain is 6.34 heterozygous SNPs/kb. Importantly, these heterozygous variants are not homogeneously distributed along the genome, but rather form blocks of high heterozygosity separated by homozygous regions (Supplementary Fig. S3). The estimated sequence divergence in the heterozygous blocks has a normal distribution (Supplementary Fig. S4), resembling the patterns previously described for *Candida* hybrids,^5^^,^^6^^,^^12^^,^^54^^,^^55^ and suggesting that these heterozygous blocks are the footprints of a past hybridization event, and not the result of the accumulation of mutations in the same lineage across time. The estimated current sequence divergence between the two haplotypes is 3.15%, which is close to the estimated sequence divergence of the parentals of *C. metapsilosis* and *C. orthopsilosis* (∼4.5%^5–7^). In a scenario of hybridization, homozygous regions represent possible tracks of LOH, an important occurrence to regain genomic stability after hybridization.^3^ In this case, we estimate that 84.41% of *C. theae* genome is covered by blocks of LOH (Table 1), a value that is similar to what was previously detected in *C. orthopsilosis* hybrid Clade 1 and in *C. albicans.*^7^^,^^54^

### 3.3. Comparison of *C. theae* environmental and clinical isolates

**Figure 2:**
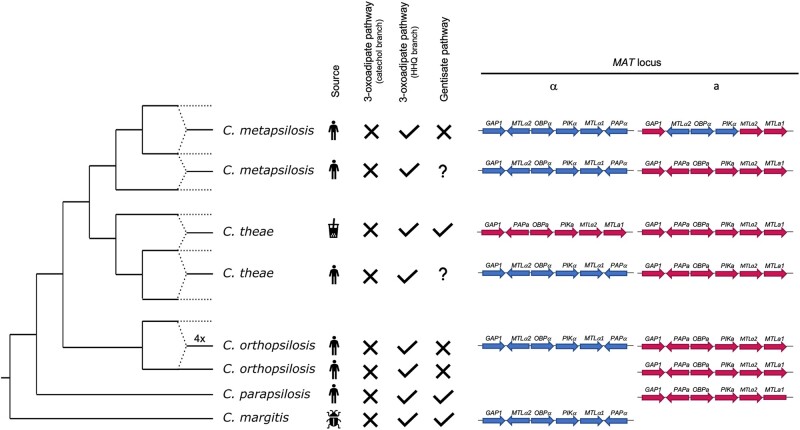
Topological reconstruction of *C. parapsilosis* species complex and summary of the features of the different lineages. Dashed lines represent hybrids’ parental lineages that have not been isolated thus far, while converging dashed lines represent the hybridization events described by this and previous studies.^5–8^ ‘4×’ indicates the occurrence of at least four independent hybridization events between the same lineages that has been described in *C. orthopsilosis.*^7^ The source indicated for each of the lineages corresponds to the source of the isolates that have been sequenced thus far. Absence or presence of the 3-oxoadipate and the gentisate pathways is indicated with a cross or a check mark, respectively, with lineages for which these features were not assessed presenting a question mark. MAT locus composition as determined by this and previous studies ^6–8^^,^^65^^,^^66^ is indicated with blue and pink arrows for *alpha* and *a* alleles, respectively. The size and the distance of the arrows is not proportional to gene size and distance but their direction represents the strand where the gene is coded. Rectangles indicate pseudogenes.

In this study, we assembled the genome of the type strain of *C. theae*, which, as above-mentioned, was isolated from a tea can.^23^ However, a recent publication reported the case of *C. theae* infection in an immunocompromised child.^24^ Taking advantage of the whole-genome sequencing data publicly available for a clinical isolate from that infection, we performed a comparative genomics analysis between the two isolates. Our results show that 98.4% of the Illumina sequencing reads of the clinical isolate aligned to the genome assembly described in this work, and that the clinical isolate presents 4.59 heterozygous SNPs/kb, thus showing an overall lower level of heterozygosity as compared with the environmental isolate (6.39 SNPs/kb). As shown in Supplementary Fig. S4, the patterns of genomic variability of the clinical isolate were also consistent with a hybrid origin for this isolate. However, in this case, we estimated 3.6% sequence divergence between haplotypes, which is higher than the 3.15% found for the type strain. Two possible scenarios could explain these discrepancies between the two isolates of the same species: (i) both *C. theae* isolates descend from the same hybridization event but the clinical isolate accumulated more mutations in the heterozygous blocks and experienced additional events of LOH; or (ii) the isolates descend from independent hybridization events. To determine which was the most plausible scenario, we compared the heterozygous positions in the intersection of heterozygous blocks of the two isolates (i.e. where both have the two ancestral haplotypes) and determined the number of shared alleles. Our results revealed that only 39.5% of the heterozygous SNPs of the environmental isolate and 19.7% of the heterozygous SNPs of the clinical isolate are shared between the two. On the other hand, still in regions that correspond to heterozygous blocks in both isolates (ancestral regions), 59.1% and 79.7% of the heterozygous positions of the environmental and the clinical isolates, respectively, are exclusive of one isolate, i.e. correspond to homozygous positions in the other one. These results are in stark contrast with what was previously found in *C. albicans* where even with a high intra-species variability, different strains shared ∼80% of their heterozygous SNPs in the ancestral block.^54^ These results suggest that, although the ITS region of both *C. theae* isolates classify them as the same species,^24^ in fact they originated from independent hybridization events between different closely related lineages (Fig. 2). This scenario resembles what was previously described in the other hybrids of the *C. parapsilosis* species complex, where multiple independent hybridization events occurred in *C. orthopsilosis* and *C. metapsilosis*,^7^^,^^8^ showing a remarkably high hybridization propensity in this clade.

### 3.4. Analysis of the MAT locus

As the Mating-type (MAT) locus can provide a better understanding of the origin of the two isolates of *C. theae*, we inspected this region in their genome assemblies. The MAT loci of *Candida* species of the CUG-Ser clade can harbour *a* and/or *alpha* idiomorphs surrounded by a conserved set of genes (Fig. 2). Although the mechanism by which hybridization occurs in these species is not fully understood, hybridization can occur through mating between cells harbouring opposite mating types. This scenario is supported by the finding of both mating type idiomorphs in hybrids of *C. orthopsilosis* and *C. metapsilosis*.^6^^,^^7^ In accordance with this expectation is the MAT locus of the *C. theae* clinical isolate, which harbours both *a* and *alpha* idiomorphs, but not the one of *C. theae* type strain, which only has MAT *a* idiomorph (Fig. 2 and Supplementary Fig. S5). Further inspection of the region surrounding *C. theae* MAT locus shows evidence for the occurrence of a recombination event in the type strain that led to LOH in this region. Therefore, we hypothesize that both hybridizations occurred between cells harbouring different MAT idiomorphs and that a subsequent introgression event erased the *alpha* alleles of the type strain. Interestingly, this is not the first instance in which such a recombination event is described in hybrids of the *C. parapsilosis* species complex. Genomic analysis of *C. metapsilosis* hybrids also revealed the existence of recombination in the MAT locus, with MAT *a* being partially replaced by MAT *alpha* alleles.^6^ To the best of our knowledge, the relevance of these recombination events leading to LOH in the MAT locus of *Candida* hybrids is not known. Nevertheless, previous studies have shown that the disruption of the MAT locus may contribute to restoring hybrids’ fertility.^56^ Further studies, exploring the mechanisms by which *Candida* species hybridize and the genomic aftermath of hybridization will be essential to clarify if the same is happening in these species.

### 3.5. Linear mitochondrial genomes

Linear mitochondrial genomes occur in several yeast species sparsely distributed in the subphylum Saccharomycotina (e.g. in the families *Debaryomycetaceae*, *Phaffomycetaceae*, *Pichiaceae*, *Saccharomycodaceae*, *Trichomonascaceae*) indicating their recurrent emergence in evolution presumably from closely related circular ancestors.^18^^,^^48^^,^^57^^,^^58^ This conclusion is further supported by three different types of telomeric structures present at the ends of linear mitochondrial DNAs, i.e. covalently closed single-stranded hairpins (Type 1), arrays of tandem repeats (Type 2) and a protein covalently bound to the 5′ termini of DNA molecules (Type 3).^18^^,^^59^^,^^60^ The Type 2 mitochondrial telomeres found in the *C. parapsilosis* species complex are of particular interest as they resemble to the telomeres at the ends of eukaryotic nuclear chromosomes and their investigation provides an insight into telomerase-independent means of telomere maintenance.^20^^,^^61^ However, the evolutionary origin of the Type 2 linear mitochondrial genomes remains elusive. Here we show that, similarly to other three members of the *C. parapsilosis* species complex, *C. margitis* and *C. theae* contain linear mitochondrial DNAs of about 25 kp long terminating with tandem repeat arrays (i.e. 2n × 549 and 2n × 553 bp, respectively) on both ends (Table 2, Supplementary Fig. S6). The mitochondrial genomes of all five species from the *C. parapsilosis* species complex share the same set of genes arranged in the same order, yet they differ by the size of the mitochondrial telomere repeat unit (Table 2). *Candidajiufengensis* and *C. pseudojiufengensis* belonging to a sister lineage of the *C. parapsilosis* species complex (Fig. 1) possess circular mitochondrial genomes with rearranged gene order compared with the *C. parapsilosis* species complex. In this study, we identified the linear mitochondrial genome also in *C. oxycetoniae*, which represents a lineage branching off before the split between the *C. parapsilosis* species complex and the lineage formed by *C. jiufengensis* and *C. pseudojiufengensis* (Fig. 1). The *C. oxycetoniae* mitochondrial genome (62.6 bp) is almost twice as long as the mitochondrial DNAs of the remaining species and, in addition to canonical mitochondrial genes, it also codes for DNA (*dpo*) and RNA (*rpo*) polymerases homologous to those carried by linear mitochondrial plasmids, which possess a protein covalently bound to the 5′ ends such as pPK2 of *Pichia kluyveri*.^62^ The presence of inverted terminal repeats as well as the *dpo* and *rpo* genes located on the opposite subtelomeric regions of the *C. oxycetoniae* linear mitochondrial DNA indicate that these terminal structures are derived from a linear plasmid. Although we did not investigate if a protein is present at the 5′ termini of DNA molecules, we assume that the linear mitochondrial DNA of *C. oxycetoniae* has Type 3 telomeres. Differences in the molecular architectures of the linear mitochondrial genomes occurring in *C. oxycetoniae* and the members of the *C. parapsilosis* species complex indicate that they emerged independently in evolution. Therefore, the search for ancestral form of the Type 2 linear mitochondrial genomes will require screening of mitochondrial genomes in additional closely related species from this clade.

**Table 2:** Characteristics of the mitochondrial genomes of *C. jiufengensis*, *C. pseudojiufengensis*, *C. margitis*, *C. theae* and *C. oxycetoniae* in comparison to what was previously described for *C. metapsilosis*, *C. orthopsilosis* and *C. parapsilosis* with indication of mitochondrial genome topology, genome size, GC content and GenBank accession numbers (for the genetic organization see Supplementary Fig. S6).

Species	Strain	Mitochondrial genome topology	mtDNA size [bp]	%GC	GenBank accession numbers and references
*C. jiufengensis*	CBS10846^T^	Circular	29 672	28.4	GU136397^48^; this study
*C. pseudojiufengensis*	CBS10847^T^	Circular	33 625	27.8	KC993179^49^; this study
*C. margitis*	CBS14175^T^	Linear, Type 2	25 333 + 2*n*× 549	23.3	This study
*C. metapsilosis* ^a^	CBS10907^T^	Linear, Type 2	23 062 + 2*n*× 620	25.1	NC_006971^16^
*C. orthopsilosis* ^a^	MCO471	Linear, Type 2	24 697 + 2*n*× 777	24.5	DQ026513^17^
*C. parapsilosis*	CBS604^T^	Linear, Type 2	30 923 + 2*n*× 738	23.8	DQ376035^17^
*C. theae*	CBS12239^T^	Linear, Type 2	25 128 + 2*n*× 553	24.3	KC993195^49^; this study
*C. oxycetoniae*	CBS10844^T^	Linear, Type 3	62 593	25.9	KC993187^49^; this study

a Note that strains with circularized (mutant) mitochondrial genomes were identified in these species.^14^

### 3.6. Assimilation of hydroxybenzenes and hydroxybenzoates

**Figure 3:**
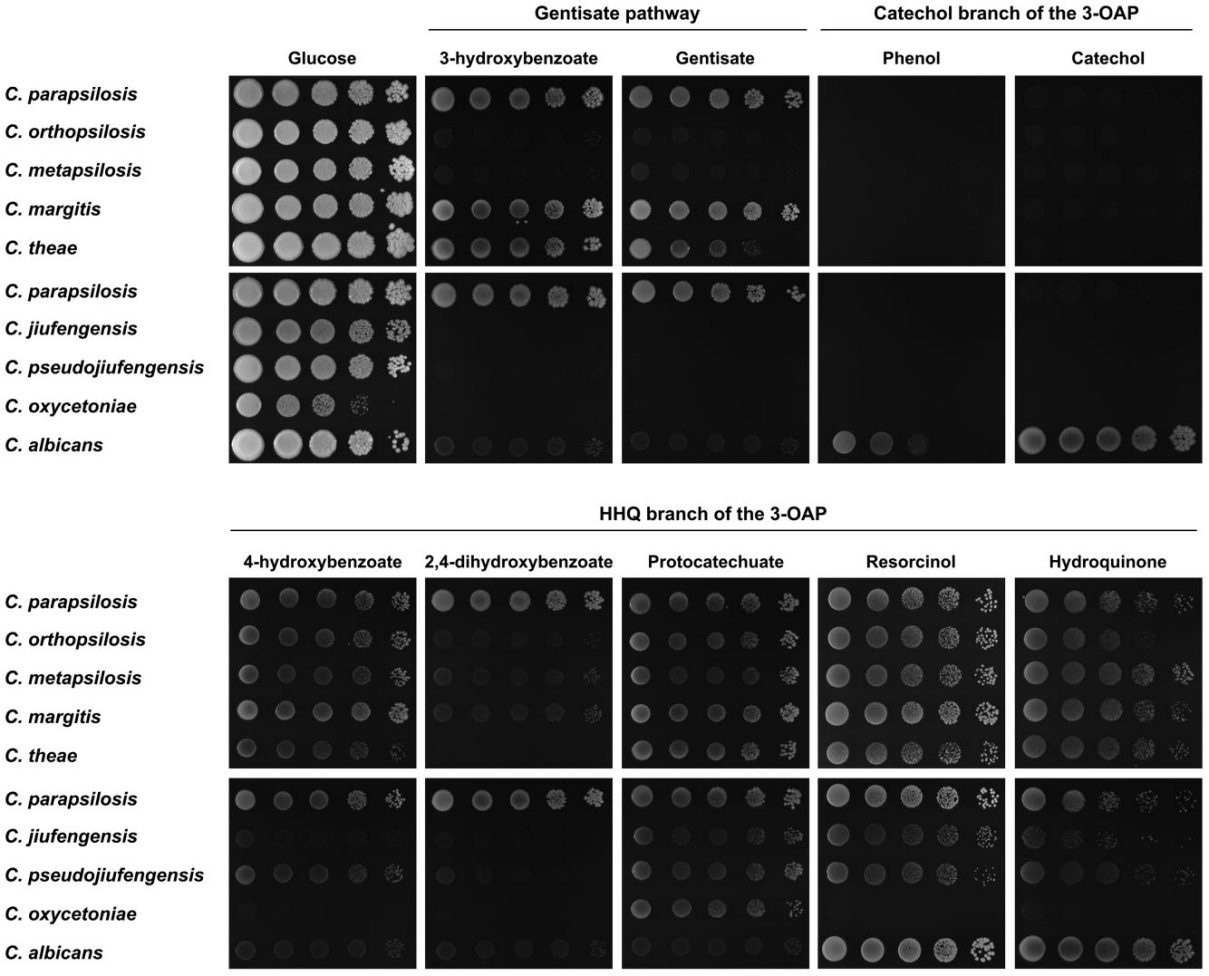
Assimilation of hydroxybenzenes and hydroxybenzoates. The cells were grown overnight in a complex liquid medium, washed with water and diluted to a concentration 6 × 10^6^ cells/ml. Serial 5-fold dilutions were spotted onto plates with synthetic media containing indicated carbon sources (see Materials and methods for details). The plates were incubated for 7 days at 28°C. The metabolic pathways involved in catabolism of corresponding substrates are indicated [i.e. the gentisate pathway and the catechol and hydroxyhydroquinone (HHQ) branches of the 3-oxoadipate pathway (3-OAP)].

Taking advantage of the orthology and paralogy relations that were inferred with the phylome reconstruction, we identified the homologs of the genes coding for the enzymes and plasma membrane transporters of the 3-oxoadipate and the gentisate pathways, as well as the transcription factors involved in their control, in *C. jiufengensis*, *C. pseudojiufengensis*, *C. oxycetoniae, C. margitis* and *C. theae* using the queries from *C. parapsilosis* and *C. albicans* (Supplementary Fig. S7, Supplementary File S3). Our results indicate that, contrary to the catechol branch of the 3-oxoadipate pathway, the HHQ branch of this pathway occurs in all these species (Fig. 2 and Supplementary Fig. S7). However, the genes coding for key enzymes of the catechol branch of this pathway (i.e. CDX1/HQD2, MCI1, MLI1, OEL1) are present only in *C. albicans*. The homologs of *C. parapsilosis* genes coding for the components of the gentisate pathway were identified in *C. margitis* and *C. theae*. Our searches also revealed that, except for MNX2 and GTF1, *C. metapsilosis* holds multiple copies of these genes (Supplementary File S3). Next, we examined the growth of these species in the synthetic media containing a hydroxybenzene or a hydroxybenzoate as a sole carbon source to verify if these pathways are functional (Fig. 3). For comparison, *C. albicans*, *C. metapsilosis*, *C. orthopsilosis* and *C. parapsilosis* were also included in this experiment. Except *C. albicans*, all examined yeasts grow in medium with protocatechuate, which confirms that the HHQ branch of the 3-oxoadipate pathway is active in these species. However, the growth of some of them is impaired on dihydroxybenzenes (*C. oxycetoniae*), 4-hydroxybenzoate (*C. jiufengensis*, *C. oxycetoniae*) and 2,4-dihydroxybenzoate (*C. jiufengensis*, *C. margitis*, *C. metapsilosis*, *C. orthopsilosis*, *C. oxycetoniae*, *C. pseudojiufengensis*, *C. theae*) pointing to a possibility that the uptake of corresponding substrates is absent or less efficient, yet functional studies need to be performed to address this hypothesis. In contrast to *C. albicans*, the other examined yeasts do not utilize phenol or catechol, which confirms the conclusion that the catechol branch is absent in these species. Our experiment also shows that, similar to *C. parapsilosis*, *C. margitis* and *C. theae* assimilate 3-hydroxybenzoate and gentisate as carbon sources, demonstrating that the gentisate pathway is active in these species. Since *C. metapsilosis* does not grow on the gentisate pathway substrates, we assume that at least some of the identified homologs are not functional in this yeast.

### 3.7. Concluding remarks

In recent years, the emergence of new pathogenic lineages has shifted the epidemiology of candidiasis.^2^ Species which so far were not considered as medically relevant are now emerging as new pathogens. Our understanding of how new pathogens emerge is very limited. In this context, the study of genomes from recently emerged species and the inference of their past evolution provides a promising research avenue. Recent studies have pointed to a role of hybridization on the emergence of new pathogens, with different hybrid lineages being described in clinical isolates, such as *C. orthopsilosis* and *C. metapsilosis*.^3^^,^^6–8^^,^^12^^,^^54^^,^^55^^,^^63^^,^^64^ Importantly, most of the parental lineages of these hybrids remain unidentified, suggesting they are never associated with infections. This has led to the hypothesis that hybridization between non-pathogenic parental species may have originated hybrid lineages with the ability to associate with and infect humans.^6^ As such, a hypothesis can only be corroborated with a comparative genomics and transcriptomics analysis between the hybrids and their respective parentals, we attempted to identify these lineages by sequencing five recently identified and closely related species, which did not correspond to clinical isolates, namely, *C. oxycetoniae*, *C. jiufengensis, C. pseudojiufengensis, C. theae* and *C. margitis.* Contrary to our expectations, none of these species corresponded to any of the unknown parental lineages, thus hampering the identification of the mechanisms underlying the possible role of hybridization on the emergence of *Candida* pathogens. However, surprisingly, the genomic patterns of *C. theae* type strain indicated that this species has a hybrid origin. Furthermore, the comparative analysis of the genome of a clinical isolate of the same species indicated that it has an independent hybrid origin from related but different parental lineages. These findings reinforce the idea that lineages of the *C. parapsilosis* clade are prone to hybridize, and that many opportunistic pathogens within this species complex are hybrids. To understand what characteristics provide these hybrids with the ability to infect humans, as opposed to their parentals, the identification of parental species and of hybrids non-associated to humans is necessary. For this, it is crucial to increase our capacity to isolate and sequence new species, including those isolated from environmental sources.

## Supplementary data

Supplementary data are available at *DNARES* online.

## Supplementary Material

dsac010_Supplementary_Data
