# Supplementary material for: Genome analysis of five recently described species of the CUG-Ser clade uncovers Candida theae as a new hybrid lineage with pathogenic potential in the Candida parapsilosis species complex
Source: DNA Res. 2022 Apr 19;29(2):dsac010. doi: 10.1093/dnares/dsac010 (PMC9046093; doi:10.1093/dnares/dsac010)
Supplement: dsac010_Supplementary_Data [file dsac010_supplementary_data.zip › Supplementary_Material_REvision.docx]

**Supplementary Material**


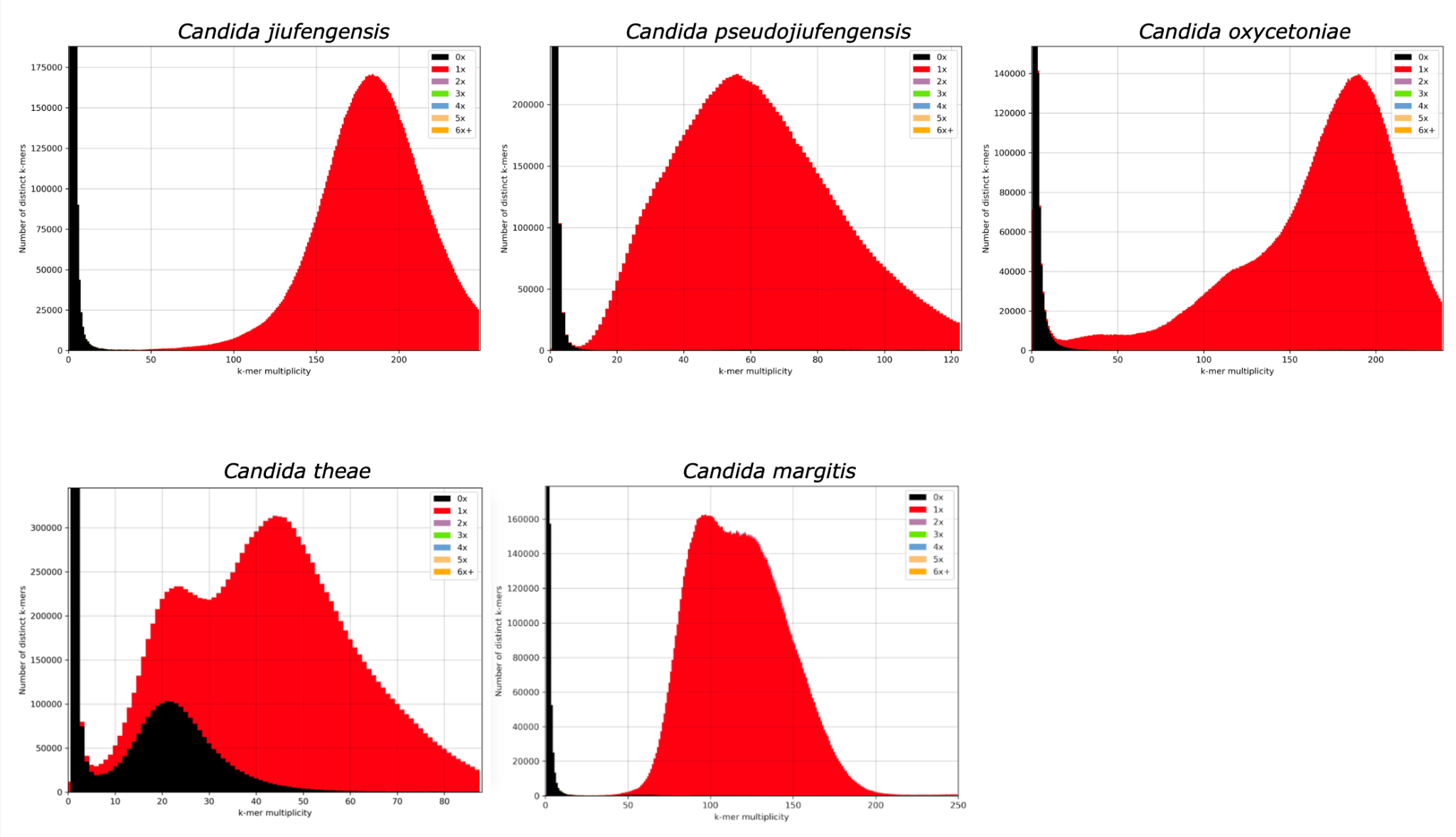


**Supplementary Figure 1.** *K-*mer frequency analysis of the sequencing reads of each of the five target species of this study in comparison to the respective genome assembly. *X*-axis represents read coverage; *y-*axis represents the number of different 27-mers observed at *x* coverage. Density of *k*-mer presence and absence in the genome assembly is represented in red and black, respectively.


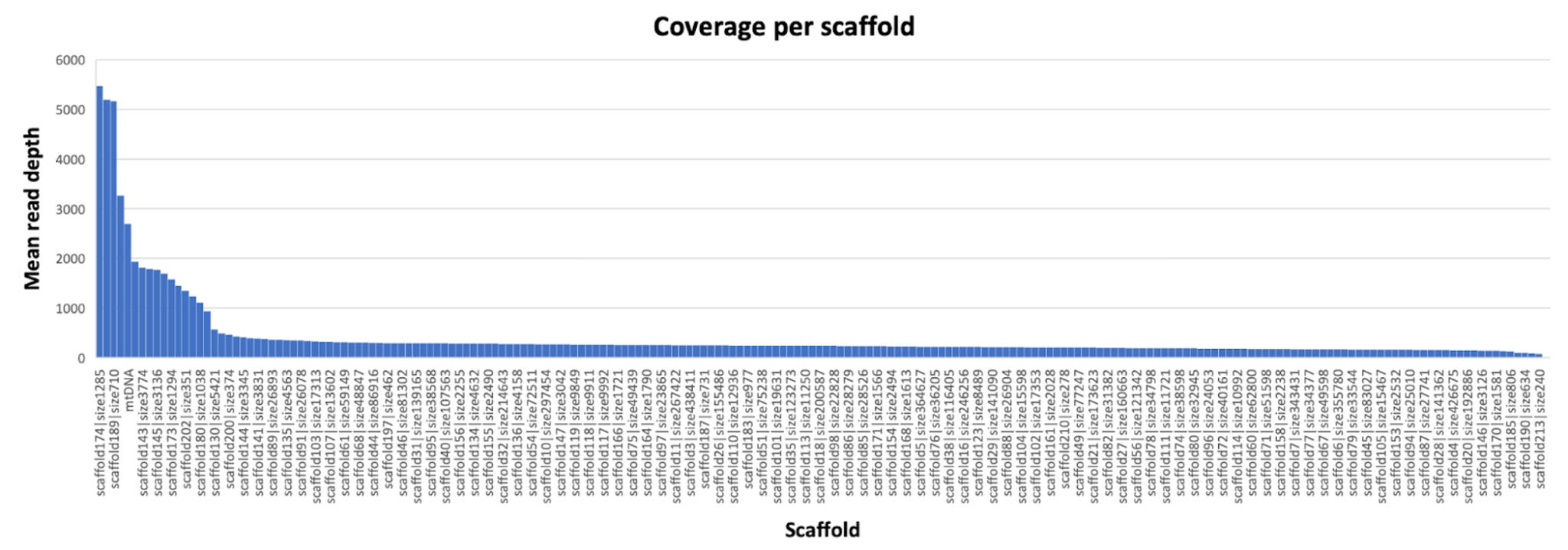


**Supplementary Figure 2.** Average read depth per scaffold of *C. margitis.*


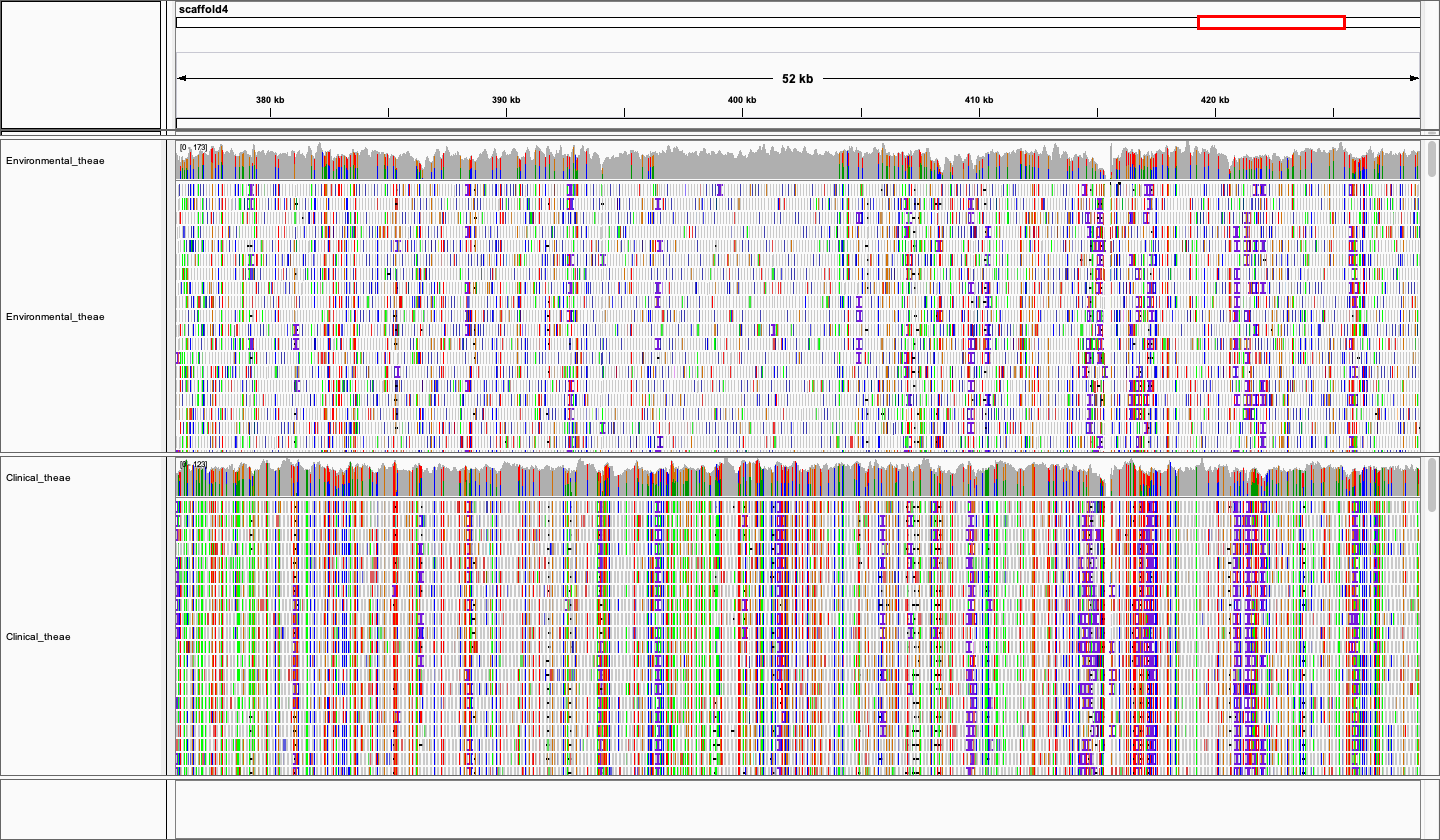


**Supplementary Figure 3.** IGV screenshot showing a 52kb region of *C. theae* scaffold 4. The first two tracks represent *C. theae* environmental isolate (type strain) coverage and read alignment on this region, and the the last two tracks represent *C. theae* clinical isolate coverage and read alignment on the same region. Colors indicate polymorphic positions. In *C. theae* type strain it is possible to visualize two heterozygous blocks separated by a block of LOH. In *C. theae* clinical isolate it is possible to observe a higher sequence divergence between the haplotypes.


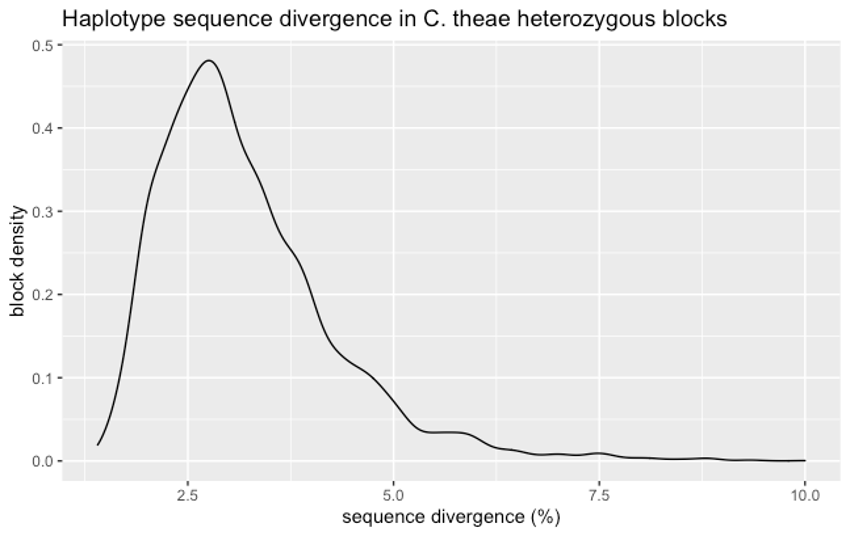


**Supplementary Figure 4.** Frequency of haplotype sequence divergence per heterozygous block determined in *C. theae*. Sequence divergence was calculated as the number of heterozygous variants called in a block, divided by the total size of the same block.


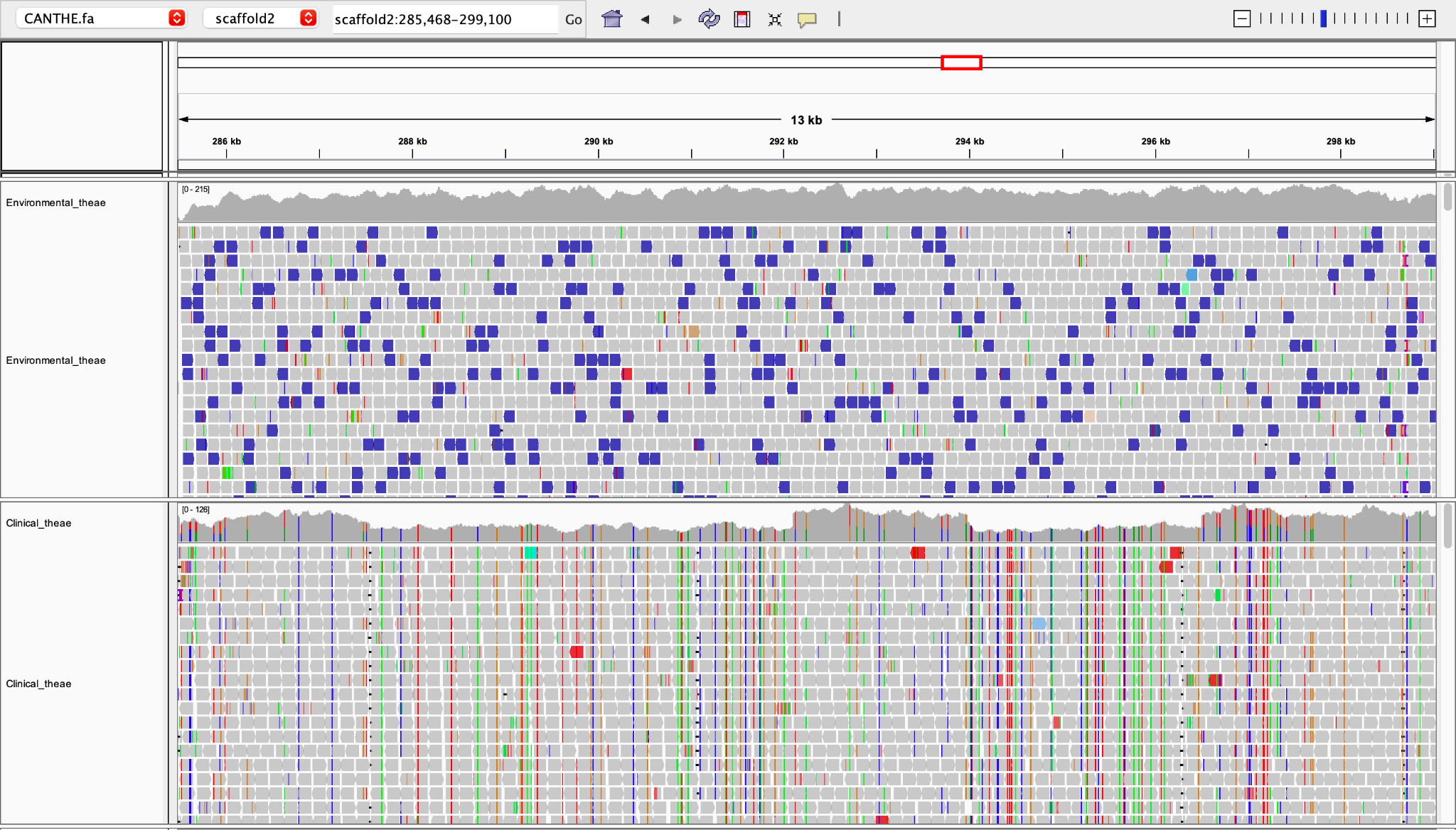


**Supplementary Figure 5.** IGV screenshot showing a 13 kb region of *C. theae* scaffold 2, which corresponds to the MAT locus. The first two tracks represent *C. theae* environmental isolate (type strain) coverage and read alignment on this region, and the last two tracks represent *C. theae* clinical isolate coverage and read alignment on the same region. Colors indicate polymorphic positions. In *C. theae* type strain, it is possible to visualize a highly homozygous region, with stable coverage, thus indicating that both chromosomes harbor the same MAT idiomorph. In *C. theae* clinical isolate, it is possible to observe a highly polymorphic region, with some genes presenting decreased coverage, i.e. half of the reads did not align. This suggests that *C. theae* clinical isolate has both MAT idiomorphs.


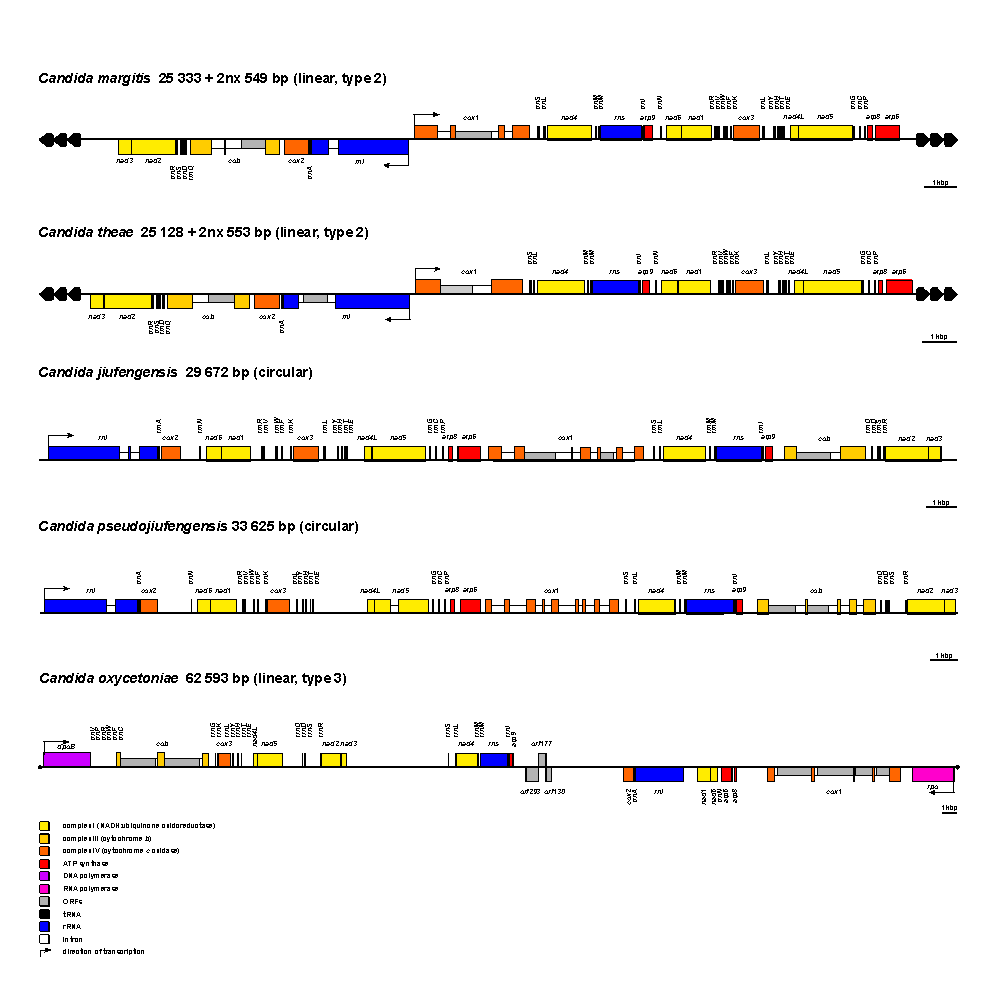


**Supplementary Figure 6.** Genetic organization of the mitochondrial genomes of *C. margitis*, *C. theae*, *C. jiufengensis*, *C. pseudojiufengensis* and *C. oxycetoniae*. Although all maps are shown in linearized form, *C. jiufengensis* and *C. pseudojiufengensis* possess circular mitochondrial genomes. The linear mitochondrial genomes of *C. margitis* and *C. theae* share similar molecular architecture with *C. metapsilosis*, *C. orthopsilosis* and *C. parapsilosis*. Note that mitochondrial telomeres of the *C. parapsilosis* species complex are different from the terminal structures of linear mitochondrial DNA in *C. oxycetoniae*.


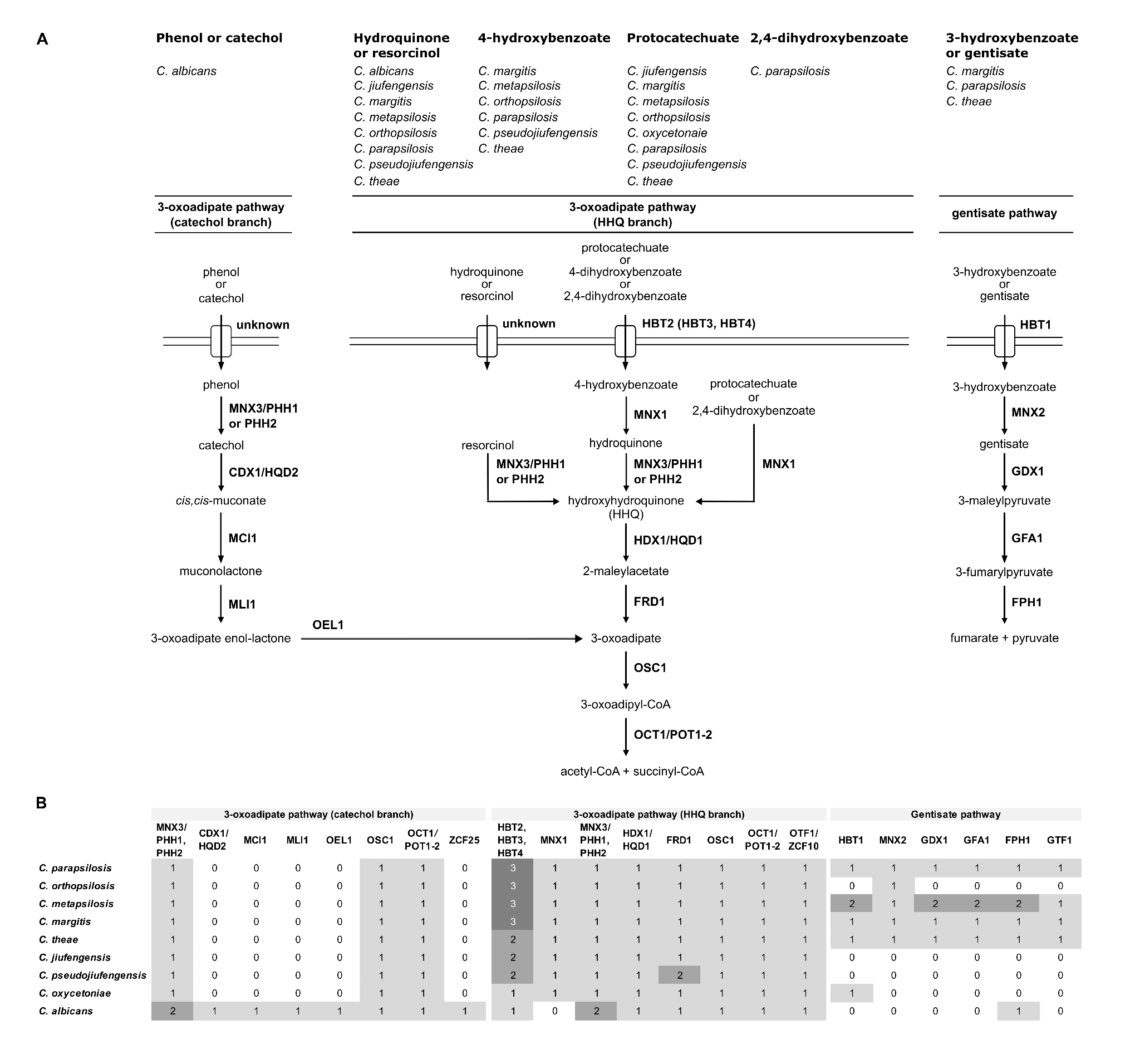


**Supplementary Figure 7.** Metabolic pathways involved in catabolism of hydroxybenzenes and hydroxybenzoates. The HHQ branch of the 3-oxoadipate pathway operates in all examined *Candida* species, however, these yeasts differ in the spectrum of assimilated substrates (Figure 3). *C. albicans* does not grow on hydroxybenzoates as it lacks an ortholog of MNX1 monooxygenase. Except for *C. albicans*, the catechol branch of this pathway is absent in all examined species. The gentisate pathway operates in *C. margitis, C. parapsilosis* and *C. theae*. Note that, despite the presence of all orthologs of corresponding genes in *C. metapsilosis*, the carbon assimilation test (Figure 3) shows that the gentisate pathway is not functional in this yeast. (A) Simplified scheme illustrating the metabolic pathways. (B) The occurrence of orthologs coding for corresponding enzymes, plasma membrane transporters (HBT1-4) and transcription factors (OTF1/ZCF10, GTF1, ZCF25) in the genome sequences of indicated *Candida* species.

**Supplementary File** **1.** List of species and respective proteome IDs used for phylome reconstruction.

**Supplementary File 2.** Phylome reconstruction and enrichment analysis of *C. jiufengensis, C. pseudojiufengensis, C. oxycetoniae, C. theae* and *C. margitis*.

**Supplementary File 3.** Gene clusters of the 3-oxoadipate and the gentisate pathways in *C. jiufengensis, C. pseudojiufengensis, C. oxycetoniae, C. margitis*, and *C. theae*.
