# Supplementary material for: Genome analysis of five recently described species of the CUG-Ser clade uncovers Candida theae as a new hybrid lineage with pathogenic potential in the Candida parapsilosis species complex
Source: DNA Res. 2022 Apr 19;29(2):dsac010. doi: 10.1093/dnares/dsac010 (PMC9046093; doi:10.1093/dnares/dsac010)
Supplement: dsac010_Supplementary_Data [file dsac010_supplementary_data.zip › SupplementaryFile_1.pdf]

Supplementary File 1. List of species and respective proteome IDs used for phylome reconstruction.

Taxon ID	Species	Proteome ID
4909	*Pichia kudriavzevii*	ISSOR.5
4922	*Pichia pastoris*	PICPA.2
4924	*Pichia stipitis*	PICST.3
4929	*Pichia guilliermondii*	PICGU.1
4932	*Saccharomyces cerevisiae*	YEAST.9
4952	*Yarrowia lipolytica*	YARLI.5
4959	*Debaryomyces hansenii*	DEBHA.5
5476	*Candida albicans*	CANAL.6
5478	*Candida glabrata*	CANGA.6
5480	*Candida parapsilosis*	CANPA.2
5481	*Diutina rugosa*	5481.2
5482	*Candida tropicalis*	CANTR.1
36911	*Clavispora lusitaniae*	CLALS.1
44093	*Trichomonascus ciferrii*	44093.2
52247	*[Candida] inconspicua*	52247.1
273372	*Candida metapsilosis*	273372.1
284811	*Ashbya gossypii ATCC 10895*	ASHGO.6
330879	*Aspergillus fumigatus*	ASPFU.4
409370	*Blastobotrys adeninivorans*	BLAAD.2
497107	*Candida oxycetoniae*	497107.2
497108	*Candida jiufengensis*	497108.3
497109	*Candida pseudojiufengensis*	497109.2
498019	*[Candida] auris*	498019.2
561895	*Candida subhashii*	561895.5
573826	*Candida dubliniensis CD36*	CANDC.1
1136231	*Candida orthopsilosis Co 90-125*	CANO9.1
1198502	*Candida theae*	1198502.2
1775924	*Candida margitis*	1775924.1
