# Supplementary material for: Genome analysis of five recently described species of the CUG-Ser clade uncovers Candida theae as a new hybrid lineage with pathogenic potential in the Candida parapsilosis species complex
Source: DNA Res. 2022 Apr 19;29(2):dsac010. doi: 10.1093/dnares/dsac010 (PMC9046093; doi:10.1093/dnares/dsac010)
Supplement: dsac010_Supplementary_Data [file dsac010_supplementary_data.zip › SupplementaryFile_2.pdf]

Supplementary File 2. Phylome reconstruction and enrichment analysis of *C. jiufengensis*, *C. pseudojiufengensis, C. oxycetoniae, C. theae* and *C. margitis*.

<u>Phylome reconstruction of *C. jiufengensis*:</u>

Phylome ID: 16

Orphan genes: 194

Enrichment analysis: Enrichment analysis for genes specifically duplicated in age 1.

Genes: 4689
Gos: 5513
Finished execution successfully.
Execution time: 0 sec.
OVER REPRESENTED TERMS
Term category: molecular_function

#overlist	term	term level	adj.pvalue	term name
1	GO:0004004	1	8.994550e-04	ATP-dependent RNA helicase activity
1	GO:0004190	1	3.331490e-04	aspartic-type endopeptidase activity
1	GO:0008026	1	3.737920e-05	ATP-dependent helicase activity
1	GO:0008186	1	1.511450e-04	RNA-dependent ATPase activity
1	GO:0022857	1	4.499660e-04	transmembrane transporter activity
1	GO:1990446	1	6.620830e-06	U1 snRNP binding
1	GO:1990447	1	6.620830e-06	U2 snRNP binding

Term category: biological_process

#overlist	term	term level	adj.pvalue	term name
1	GO:0000348	1	6.723760e-05	mRNA branch site recognition
1	GO:0008380	1	8.994550e-04	RNA splicing
1	GO:0010468	1	3.737920e-05	regulation of gene expression
1	GO:1903241	1	6.620830e-06	U2-type prespliceosome assembly

<u>Phylome reconstruction of *C. pseudojiufengensis*:</u>

Phylome ID: 404

Orphan genes: 282

Enrichment analysis: Enrichment analysis for genes specifically duplicated in age 1.

Genes: 4639
Gos: 5504
Finished execution successfully.
Execution time: 1 sec.
OVER REPRESENTED TERMS
Term category: molecular_function

#overlist	term	term level	adj.pvalue	term name
1	GO:0004190	1	5.603760e-08	aspartic-type endopeptidase activity

Term category: cellular_component

#overlist	term	term level	adj.pvalue	term name
1	GO:0005775	1	1.007880e-04	vacuolar lumen
2	GO:0005829	1	9.426170e-04	cytosol

Term category: biological_process

#overlist	term	term level	adj.pvalue	term name
1	GO:0006508	1	3.338200e-04	proteolysis

<u>Phylome reconstruction of *C. oxycetoniae*:</u>

Phylome ID: 960

Orphan genes: 221

Enrichment analysis: No significant enrichment was observed.

Phylome reconstruction of *C. theae*:

Phylome ID: 866

Orphan genes: 109

Enrichment analysis: There was no particular enrichment.

Phylome reconstruction of *C. margitis*:

Phylome ID: 423

Orphan genes: 33

Enrichment analysis: Enrichment analysis for genes specifically duplicated in age 1.

Genes: 3158
Gos: 1108
Finished execution successfully.
Execution time: 0 sec.

OVER REPRESENTED TERMS
Term category: molecular_function

#overlist	term	term level	adj.pvalue	term name
1	GO:0005515	1	6.944300e-05	protein binding
1	GO:0005524	1	5.125490e-22	ATP binding
1	GO:0042626	1	1.162760e-16	ATPase activity, coupled to transmembrane movement of substances

Term category: cellular_component

#overlist	term	term level	adj.pvalue	term name
1	GO:0016021	1	8.413220e-07	integral component of membrane

Term category: biological_process

#overlist	term	term level	adj.pvalue	term name
1	GO:0055085	1	1.123870e-11	transmembrane transport
